# Assessing the antioxidant properties of Naringin and Rutin and investigating their oxidative DNA damage effects in breast cancer

**DOI:** 10.1038/s41598-024-63498-7

**Published:** 2024-07-03

**Authors:** Badhe Pravin, Vivek Nanaware, Badhe Ashwini, Gezahign Fentahun Wondmie, Yousef A. Bin Jardan, Mohammed Bourhia

**Affiliations:** 1 Swalife Biotech Ltd Unit 3D North Point House, North Point Business Park, Cork, Ireland; 2 Swalife Labs Ltd, Uxbridge, UK; 3 Centre for Drug Discovery and Development, Sinhgad College of Pharmacy, Pune, India; 4 Department of Biology, Bahir Dar University, P.O.Box 79, Bahir Dar, Ethiopia; 5 Department of Pharmaceutics, College of Pharmacy, King Saud University, P.O. Box 11451, Riyadh, Saudi Arabia; 6 Department of Chemistry and Biochemistry, Faculty of Medicine and Pharmacy, Ibn Zohr University, 70000 Laayoune, Morocco

**Keywords:** DNA damage response, Naringin, Rutin, Cancer therapy, 2DD normal human fibroblast cells, Quiescent fibroblast cells, Selective cytotoxicity, Antioxidant activity, Biochemistry, Biotechnology, Drug discovery

## Abstract

This work examines the capacity of Naringin and Rutin to influence the DNA damage response (DDR) pathway by investigating their interactions with key DDR proteins, including PARP-1, ATM, ATR, CHK1, and WEE1. Through a combination of in silico molecular docking and in vitro evaluations, we investigated the cytotoxic and genotoxic effects of these compounds on MDA-MB-231 cells, comparing them to normal human fibroblast cells (2DD) and quiescent fibroblast cells (QFC). The research found that Naringin and Rutin had strong affinities for DDR pathway proteins, indicating their capacity to specifically regulate DDR pathways in cancer cells. Both compounds exhibited preferential cytotoxicity towards cancer cells while preserving the vitality of normal 2DD fibroblast cells, as demonstrated by cytotoxicity experiments conducted at a dose of 10 µM. The comet experiments performed particularly on QFC cells provide valuable information on the genotoxic impact of Naringin and Rutin, highlighting the targeted initiation of DNA damage in cancer cells. The need to use precise cell models to appropriately evaluate toxicity and genotoxicity is emphasized by this discrepancy. In addition, ADMET and drug-likeness investigations have emphasized the pharmacological potential of these compounds; however, they have also pointed out the necessity for optimization to improve their therapeutic profiles. The antioxidant capabilities of Naringin and Rutin were assessed using DPPH and free radical scavenging assays at a concentration of 10 µM. The results confirmed that both compounds have a role in reducing oxidative stress, hence enhancing their anticancer effects. Overall, Naringin and Rutin show potential as medicines for modulating the DDR in cancer treatment. They exhibit selective toxicity towards cancer cells while sparing normal cells and possess strong antioxidant properties. This analysis enhances our understanding of the therapeutic uses of natural chemicals in cancer treatment, supporting the need for more research on their mechanisms of action and clinical effectiveness.

## Introduction

Cellular DNA is subject to numerous physical and chemical alterations, which can negatively impact the accuracy of genetic information transfer. The causes of such harm encompass both internal physiological processes and external environmental exposures. Exogenous factors, such as ionizing radiation emitted by X-rays and cosmic rays, ultraviolet (UV) light, and oxidative agents caused by environmental pollution, initiate several types of DNA damage^1–3^. These external sources pose a challenge to the cell's systems for repairing DNA, resulting in disturbances to the integrity of the genome, which is crucial for the cell's survival and function.

Simultaneously, internal causes of DNA damage, such as mistakes in DNA replication, reactive oxygen species (ROS) produced from regular metabolic activities, and perturbations in cellular signaling pathways, highlight the complex equilibrium inside cells to preserve genomic integrity^4,5^. The interaction between these internal and external factors is crucial, with oxidative stress—caused by an imbalance between the production of reactive oxygen species (ROS) and the body's ability to remove these reactive substances or repair the resulting harm—being a significant factor in the development of various diseases, particularly cancer^6^.

The widespread occurrence of DNA damage resulting from both internal cellular processes and external environmental influences poses a constant risk to the stability of the genome. The cellular apparatus responsible for DNA repair diligently strives to correct these injuries. However, malfunctions in this system can result in mutations, chromosomal abnormalities, and eventually, the demise of cells or the development of malignancies^7^. This emphasizes the crucial function of DNA damage response (DDR) systems in maintaining the survival of cells and organisms.

Furthermore, the accumulation of free radicals, which are produced as a result of both external factors and regular metabolic processes, presents a substantial threat to essential cellular elements. Contemporary lifestyle variables, such as unhealthy eating habits, lack of exercise, and tobacco consumption, worsen the creation of these harmful substances, making the cellular environment more complex and adding to the development of chronic illnesses, including cancers^8–10^.

The capacity of human cells to withstand the constant attack from both internal and external stimuli is essential for maintaining the integrity of the genome, which is critical for preventing illnesses such as cancer and slowing down the aging process. Every day, our cells encounter numerous instances of DNA damage that have the potential to impair crucial activities like replication and transcription. This highlights the need for strong DNA repair systems^10,11^. The DNA damage response (DDR) coordinates an intricate network of repair pathways, including nucleotide excision repair (NER), mismatch repair (MMR), base excision repair (BER), and double-strand break repair (DSBs), to combat various types of damage. The efficacy of this mechanism is crucial for repairing DNA lesions, which is essential for preserving cellular integrity and avoiding mutations that may lead to cancer or contribute to the aging process^12–15^. Gaining a comprehensive understanding of and improving these repair processes is crucial for developing precise therapies to combat genomic instability and its related hazards.

The cellular response to DNA damage is dependent on a complex network involving poly (ADP-ribose) polymerase (PARP-1), checkpoint kinase 1 (CHK1), ataxia-telangiectasia mutated (ATM), ataxia-telangiectasia and Rad3-related (ATR), and WEE1 (a crucial checkpoint kinase for mitotic events). PARP-1 is essential for repairing DNA strand breaks, while CHK1, in conjunction with RAD51, is critical for maintaining genomic integrity during periods of stress^16–18^. The kinases ATM and ATR, together with their associated proteins, play a crucial role in identifying DNA damage and initiating the necessary repair mechanisms to maintain the integrity of the cell cycle^19,20^. WEE1 has a role in regulating the cell cycle by modulating the initiation of cell division, making it a potential target for treatments in cancer therapy^21,22^. This network highlights the intricate nature of DDR and the several potential approaches for cancer therapy.

The inquiry into the therapeutic properties of plant-derived chemicals, particularly Naringin and Rutin, arises from their well-established functions in regulating pathways crucial to the advancement of diseases. The relevance of these phytochemicals is highlighted by their adaptability in addressing a range of illnesses, including cancers, metabolic disorders, and cardiovascular problems^23,24^. These chemicals, Naringin found in citrus fruits and Rutin found in many fruits, vegetables, and drinks, are known for their prevalence in common dietary sources. They provide a convenient way to use natural anticancer and protective mechanisms^25–27^.

Rutin, known for its powerful antioxidant ability, is gaining attention as a molecule of interest because of its many pharmacological effects, which include regulating inflammation, managing diabetes, protecting blood vessels, exhibiting antimicrobial activity, and most importantly, possessing anticancer capabilities. Rutin's capacity to impede tumor development, halt the cell cycle, and trigger apoptosis in several cell types presents a hopeful approach to cancer treatment, capitalizing on its inherent presence and wide-ranging advantages^26^.

Similarly, Naringin, derived from traditional medicine and widely found in citrus fruits, has substantial therapeutic promise. The chemical has a wide range of therapeutic properties, including antioxidant, anticancer, antiviral, antibacterial, anti-inflammatory, anti-adipogenic, and cardioprotective actions, demonstrating its effectiveness in treating many conditions^27^. The choice of Naringin and Rutin for this study is based on their proven effectiveness in laboratory studies, which have shown their ability to regulate important biological processes related to cancer development and other diseases. This provides a basis for investigating their potential as therapeutic agents.

The choice to investigate the use of Naringin and Rutin in cancer treatment is based on a thorough combination of computational and empirical research methods. By using PyRx and AutoDock Vina, our molecular docking analyses predict strong binding affinities between these natural chemicals and key proteins involved in the DNA damage response (DDR) pathway. Simultaneously, the Swiss ADME and pkCSM ADMET tools have offered valuable pharmacokinetic profiles, supporting the medicinal potential of Naringin and Rutin. The experimental phase involves the use of human dermal fibroblast (2DD) cells and cancer cells to assess the cytotoxic and antioxidant capabilities of these flavonoids. Utilizing a high alkaline comet test provides insight into oxidative DNA damage, allowing for differentiation between the resistance of normal cells and the vulnerability of cancer cells to DNA damage. The combination of in silico predictions and in vitro trials emphasizes the potential therapeutic benefits of Naringin and Rutin in cancer treatment. This also highlights the synergistic effects that may be achieved by integrating computational analysis with biological research

## Material and methods

Modified Eagle’s Minimal Essential Medium (DMEM), phosphate buffer (pH 7.4), trypsin-EDTA, penicillin, streptomycin, glutamine, fetal bovine serum (FBS), dimethyl sulfoxide, trypan blue, and thiazolyl blue tetrazolium bromide were obtained from Sigma-Aldrich (UK). Human dermal fibroblasts (2DD) and breast cancer cells (MDA-MB-231) were purchased from the Health Protection Agency culture collection. Disodium phosphate (Na_2_HPO_4_), monopotassium phosphate (KH_2_PO_4_), and ethylenediaminetetraacetic acid (EDTA) were purchased from Sigma-Aldrich (UK).

Deoxyribose, ethylenediaminetetraacetic acid (EDTA), L-ascorbic acid, iron(III) chloride hexahydrate, thiobarbituric acid, trichloroacetic acid, hydrogen peroxide, sodium hydroxide (NaOH), potassium nitrite, manganese dioxide, diethylenetriamine pentaacetate (DTPA), sodium chloride (NaCl), potassium chloride (KCl), Evans blue, nicotinamide adenine dinucleotide, nitro blue tetrazolium, phenazine methosulphate, sodium nitroprusside, sulphanilamide, glacial acetic acid, napthylethylenediamine dihydrochloride (NED), and Griess agent were also purchased from Sigma-Aldrich (UK). All chemicals used were of analytical grade

### In silico studies

In silico study is a computational method used to study chemical compound databases to identify molecules with desired biological activity. AutoDock Vina, implemented on PyRx 0.8, was used in this study to calculate the binding energies^28^. High throughput Virtual Screening (HTVS) programs through PyRx software with graphical user interfaces (GUIs) that employ AutoDock for predicting receptor-ligand interactions are beneficial for the comparison of ligands. AutoDock Vina is a software that works on the premise of empirical scoring functions and also calculates the grid maps automatically.

### Ligands selection and preparation

The structures of the biomolecules Naringin and Rutin, as well as the standards Olaparib, AZD0156, AD6738, AZD7762, and AZD1775, were retrieved using the PubChem compound database. All the 3D structures of the bioactive molecules were obtained in structural data format (SDF). The retrieved biomolecules were then minimized using Open Babel, with the uff force field and conjugate gradients as the optimization algorithm, available in PyRx 0.8.

### Preparation of macromolecule

DNA damage response proteins were retrieved from the PDB website (https://www.rcsb.org) and analyzed using Discovery Studio 4.0. The retrieved molecules were initially complexed with water molecules and hetero-atoms. To avoid docking interference, these hetero-atoms and water molecules were removed using Discovery Studio 4.0. Hydrogen atoms were added, and the molecules were saved in PDB format. The specific PDB files retrieved included PARP protein (PDB ID: 4UND), ATM (PDB ID: 5NP1), ATR (PDB ID: 4IGK), CHK1 (PDB ID: 2YEX), and WEE1 (PDB ID: 7N3U).

### Ligand protein docking

Docking was performed by using PyRx 0.8. After the completion of docking, auto dock preferences were obtained for both ligand and target in PDBQT format. The docking of Protein and ligand was viewed using Discovery studio 4.0 and ligand-protein interaction was analyzed. The pose of minimum binding energy was chosen as the best interaction.

### ADMET and drug-likeness predictions of ligand

The pharmacokinetic properties—such as absorption, distribution, metabolism, excretion, and toxicity (ADMET)—of bioactive molecules play an important role in drug development. Therefore, all possible pharmacokinetic parameters and toxicity of the selected biomolecules were predicted using Swiss ADME^29^ and pkCSM^30^.

### Cell culture

Human dermal fibroblast (2DD) and human breast adenocarcinoma (MDA-MB-231) were grown with the help of culture media (Table 1).

**Table 1:** Composition of cell culture after modification adopted from^31^.

Sr No	Name of ingredient	Quantity
1	Dulbecco's Modified Eagle's Medium with a high glucose content (4.5 g l − 1)	500 mL
2	10% v/v Foetal Bovine serum	56.5 mL
3	100 U ml − 1 Penicillin and 10µg ml − 1 streptomycin	6 mL
4	1% Glutamine	6 mL
5	1% non-essential amino acids	6 mL

All ingredients listed in Table 1 were pre-warmed to 37 °C before preparing the medium. All materials placed in the cabinet were sprayed with Bioguard. Hand gloves were used while working in the cabinet to maintain aseptic conditions.

Ingredients 2–5 from Table 1 were measured and added to a bottle of Dulbecco's Modified Eagle's Medium (DMEM). The medium was filtered using a 0.2 µm filter. Cell vials were removed from the nitrogen freezer and placed in a 37 °C water bath to rapidly defrost the suspension. Cells were plated in 90 mm petri dishes and placed in a humidified incubator at 37 °C with 5% carbon dioxide. The medium was changed on Tuesdays and Fridays. The cells were passaged twice a week.

To pass the cells, the medium was removed, and the plate with the culture was washed using Versene (containing 0.02% KCl (w/v), 0.8% NaCl (w/v), 0.02% KH_2_PO_4_ (w/v), 0.0115% Na_2_HPO_4_ (w/v), and 0.2% EDTA (w/v)). The cultures were then treated with a solution of 0.25% trypsin: Versene (1:10, v:v) to detach the cells from the tissue culture flasks (approximately 3–5 minutes). The effect of trypsin was neutralized by adding an equal volume of DMEM medium. This cell suspension was centrifuged at 1000 rpm for 5 minutes. The supernatant was removed, and the cells were resuspended in a known volume of fresh medium^32^.

### Cell counting

Before conducting any cell-based assays, cells were enumerated using the hemocytometer cell counting method.

## Cytotoxicity assay

### MTT assay

The MTT assay was performed on MDA-MB-231 cells to evaluate the cytotoxicity of the tested molecules. The molecules were applied in serial dilution. Cytotoxicity was determined by plotting a graph of cell viability versus concentration.

$${\text{Cell}}\;{\text{viability}}\;{\text{formula}} = \left( {{\text{Absorbance}}\;{\text{of}}\;{\text{ sample}}/{\text{Absorbance}}\;{\text{of}}\;{\text{positive}}} \right){\text{x1}}00$$

Cells were seeded on 96 well plates at a final concentration of approximately 1.5 x 10^4^ cells per 200 µl medium per well 24 hours before the assay. 96 well plates with cell suspensions were then incubated at 37 °C for 24 hours.

After 24 hours, the cell media was removed, and the cells were treated with different concentrations of the molecules, followed by incubation at 37 °C for an additional 24 hours.

After 24 hours 20 µL MTT (5mg/mL) dye solution in PBS was added to 96 well plates and was incubated with cells for 3 hours at 37 °C. After three hours the media containing MTT was removed and the plates were washed with 100 µl of PBS. After washing with PBS the solution of DMSO (200 µl) was added to the wells and kept on a shaker for 5–10 minutes. The absorbance was measured at 580nm using a BMG LABTECH microplate reader^33^.

### DPPH assay

The DPPH assay was conducted using a microplate reader (BMG LABTECH Instrument). Each reaction mixture in the 96 wells consisted of molecule solutions, aqueous methanol solution, and 70% ethanol containing DPPH radicals as a blank."The mixture was left to stand for 60 min in the dark. The reduction of the DPPH radical was determined by measuring the absorption at 517 nm^34^.

### Hydroxyl radical scavenging activity assay

The assay was adopted from^35^ with slight modifications. The procedure was initially performed in test tubes and then transferred to a 96-well plate for absorbance measurement.

First, a mixture containing 3.6 mM deoxyribose, 0.1 mM EDTA, 0.1 mM L-ascorbic acid, 1 mM H_2_O_2_, and 0.1 mM iron(III) chloride hexahydrate (FeCl_3_·6H_2_O) was prepared. Then, 10 µM of the test molecules were added to this mixture, and the volume was adjusted to 1 mL with 25 mM phosphate buffer (pH 7.4). This mixture was incubated for 1 h at 37 °C.

After incubation, 500 µL of 1% thiobarbituric acid and 500 µL of 1% trichloroacetic acid were added to the mixture. The mixture was then heated in a water bath at 80 °C for 20 min and subsequently cooled. Absorbance was measured at 532 nm. The control reaction contained no test sample.

### Peroxynitrite scavenging activity assay

The peroxynitrite scavenging activity assay consists of two main parts: the synthesis of peroxynitrite and the measurement of its scavenging activity using the Evans blue bleaching assay^36^. Peroxynitrite was synthesized by mixing an acidic solution of 0.7 M hydrogen peroxide (H_2_O_2_) with an equal volume of 0.6 M potassium nitrite in an ice bath. Subsequently, an equal volume of ice-cold 1.2 M sodium hydroxide (NaOH) was added to the mixture. Granular manganese dioxide, prewashed with 1.2 M NaOH, was used to remove excess H_2_O_2_. The reaction mixture was then stored at − 20 °C for 12 h.

For the measurement of peroxynitrite scavenging activity, the Evans blue bleaching assay was performed. The reaction mixture contained 0.1 mM diethylenetriaminepentaacetic acid (DTPA), 90 mM sodium chloride (NaCl), 5 mM potassium chloride (KCl), and 12.5 μM Evans blue. The synthesized peroxynitrite from the first step and 10 µM of the test molecules were added to this reaction mixture. The final volume was adjusted to 1 mL with 50 mM phosphate buffer (pH 7.4). The reaction mixture was incubated at 25 °C for 30 min, and the absorbance was measured at 611 nm. The percentage of peroxynitrite (ONOO^−^) scavenging was calculated by comparing the absorbance of the test samples with that of the blank samples.

### Superoxide radical scavenging activity assay

Superoxide radical scavenging activity was measured using a non-enzymatic system involving nicotinamide adenine dinucleotide (NADH), nitroblue tetrazolium (NBT), and phenazine methosulfate (PMS) as reported by^37^. This assay evaluates the ability of superoxide radical scavengers to inhibit the reduction of NBT by NADH in the presence of PMS, which reduces NBT to a purple formazan.

In this assay, 50 μM NBT in 20 mM phosphate buffer (pH 7.4) was added to 1 mL of NADH solution (73 μM NADH in 20 mM phosphate buffer, pH 7.4) containing 10 μM of the test molecules. The reaction was initiated by adding 15 μM PMS, and the absorbance was measured at 560 nm. The percentage of superoxide radical scavenging was calculated by comparing the absorbance of the test samples with that of the blank samples.

### Nitric oxide scavenging activity assay

Nitric oxide is generated at a physiological temperature from aqueous sodium nitroprusside (SNP) solution, which reacts with oxygen to produce nitrite ions detectable by the Griess-Ilosvoy reaction^36^. The reaction mixture contained 10 mM SNP in 20 mM phosphate buffer (pH 7.4) and 10 μM of the test molecules, in a final volume of 3 mL. After incubation for 150 min at 25 °C, 1 mL of sulfanilamide (0.33% in 20% glacial acetic acid) was added to 0.5 mL of the incubated solution and allowed to stand for 5 min. Then, 1 mL of NED (0.1% w/v) was added, and the mixture was incubated for 30 min at 25 °C. The absorbance was measured at 540 nm.

The percentage of nitric oxide scavenging was calculated by comparing the absorbance of the test samples with that of the blank samples. In this assay, the pink chromophore generated during the diazotization of nitrite ions with sulfanilamide and subsequent coupling with NED was measured.

### Single-cell gel electrophoresis (COMET) assay

The cells were cultivated in small Petri dishes using standard cell culture techniques for one week. 2DD cells were cultured in a low serum solution containing 0.5% serum. These cells were then plated and allowed to remain undisturbed for 7 days to induce quiescence and transform them into fibroblast-like cells. MDA-MB-231 cells were cultured according to previously established protocols. On the day of the experiment, the medium was removed, and the cells were washed twice with PBS. The cells were exposed to the test molecules for one hour. Following this incubation, the cells were rinsed twice with preheated PBS. Trypsin–EDTA was added to cover the entire monolayer of cells, and the cells were incubated at 37 °C for 2 min or until they detached upon tapping. To neutralize the trypsin, 2 mL of complete medium containing fetal bovine serum was added. The cells were transferred to a centrifuge tube and mixed with ice-cold 1X PBS to achieve a concentration of 1.5 × 10^5^ cells per milliliter.

The comet assay was performed to assess DNA damage. Cells were embedded in agarose on slides, lysed with detergent and high salt, and the DNA was immobilized for subsequent electrophoresis. The assay was modified to detect oxidative base damage by incorporating Formamidopyrimidine DNA glycosylase (FPG) enzyme. This enzyme possesses glycosylase activity to remove damaged bases and AP lyase activity to convert AP sites into breaks, allowing for the detection of additional strand breaks caused by oxidized bases.

Two sets of slides were prepared: one with FPG enzyme and one without. Each slide with FPG enzyme received 75 µL of FPG enzyme solution (1:75 FPG in FPG Flare reaction buffer). The slides were incubated at 37 °C for 60 min. Meanwhile, 2 L of alkali solution (pH > 13) were prepared by mixing 500 mM EDTA (2 mL) and NaOH (8 g) in 1 L of deionized water, and this solution was stored in a cold room at 2–8 °C. After incubation, the slides were transferred to a Coplin jar containing the alkali solution and incubated for 30 min at room temperature in the dark, with the solution changed once.

After alkali treatment, the slides were placed in a horizontal electrophoresis chamber filled with chilled alkali solution, ensuring the slides were just covered. Electrophoresis was performed at 22 V for 30 min. The slides were then dried overnight at 37 °C to bring all cells to a single plane, facilitating observation. The dried slides could be stored at room temperature with desiccant until scoring. For staining, 100 µL of diluted SYBR Green I was added to each circle of dried agarose, and the slides were refrigerated for 15–30 min before imaging.

This assay modification, incorporating FPG enzyme, enhances the detection of oxidative base damage by creating additional strand breaks from oxidized bases, allowing for a more comprehensive assessment of DNA damage^33^.

## Results

### Determining the binding affinities of Naringin and Rutin with DDR proteins

We used PyRx to conduct in silico analyses and assess the binding affinities of Naringin and Rutin with important proteins in the DNA damage response (DDR) pathway. The proteins analyzed consist of PARP-1 (poly (ADP-ribose) polymerase), ATM (ataxia-telangiectasia mutated), ATR (ataxia-telangiectasia and Rad3-related), CHK1 (Checkpoint Kinase 1), and WEE1 (Table 2).

**Table 2:** DDR protein binding affinity with Naringin and Rutin.

Sr No	Target proteins	Binding affinity		
		Naringin	Rutin	Standard
1	PARP-1(poly (ADP-ribose) polymerase)	− 10.7	− 12.4	− 12.4
2	ATM (ataxia-telangiectasia mutated)	− 10.5	− 10.3	− 9
3	ATR (ataxia-telangiectasia and Rad3-related)	− 9.1	− 8.8	− 8.3
4	CHK1(Checkpoint Kinase 1)	− 8.9	− 6.9	− 8.6
5	WEE1	− 11.1	− 9.1	− 10.3

The investigation demonstrates that Naringin exhibits a strong ability to bind to ATM, ATR, CHK1, and WEE1. In contrast, Rutin has a strong affinity specifically for PARP-1, ATM, and ATR.

### Visual representation of binding interactions

**Figure 1:**
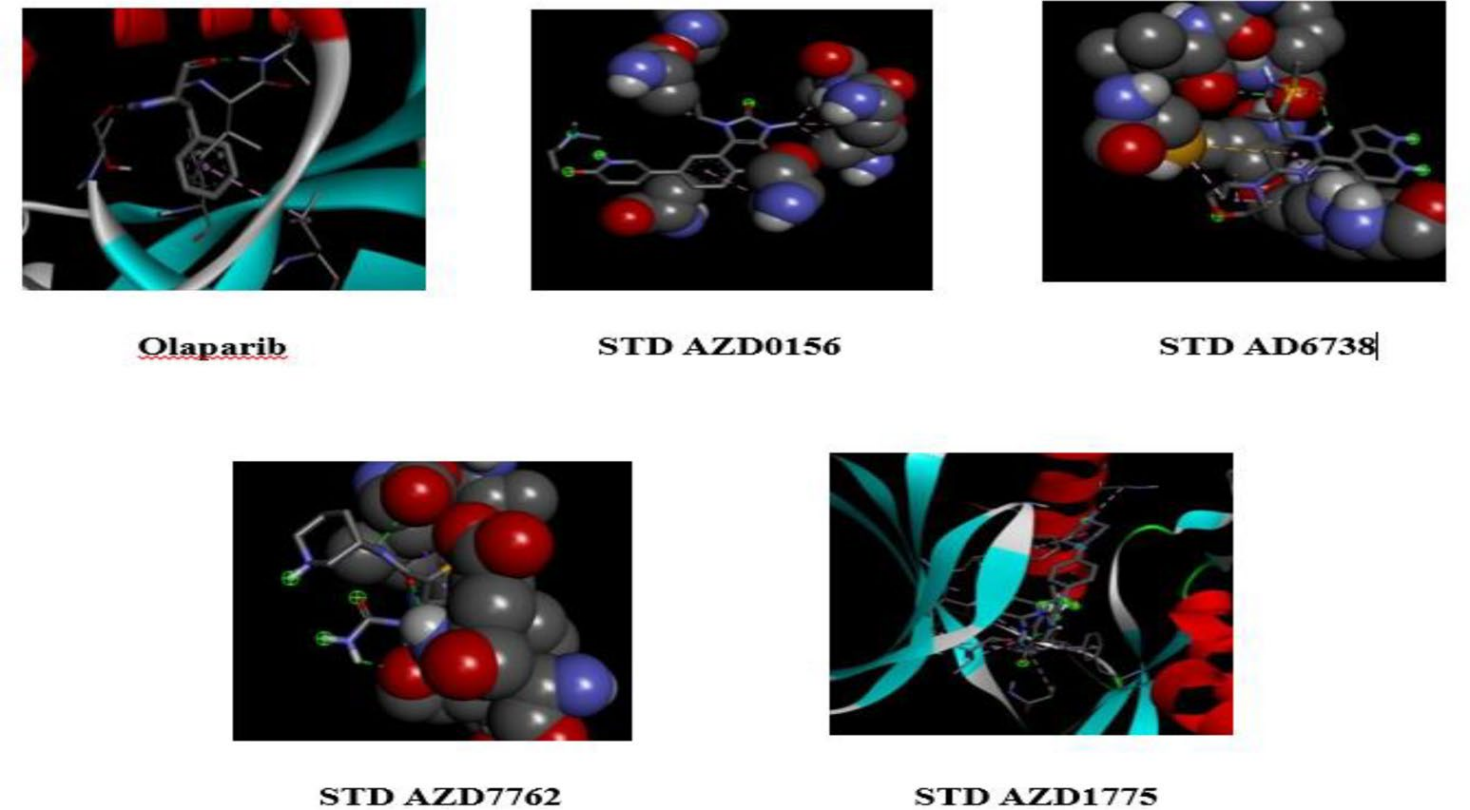
3D structure of standards used in Pyrx based molecules docking.

**Figure 2:**
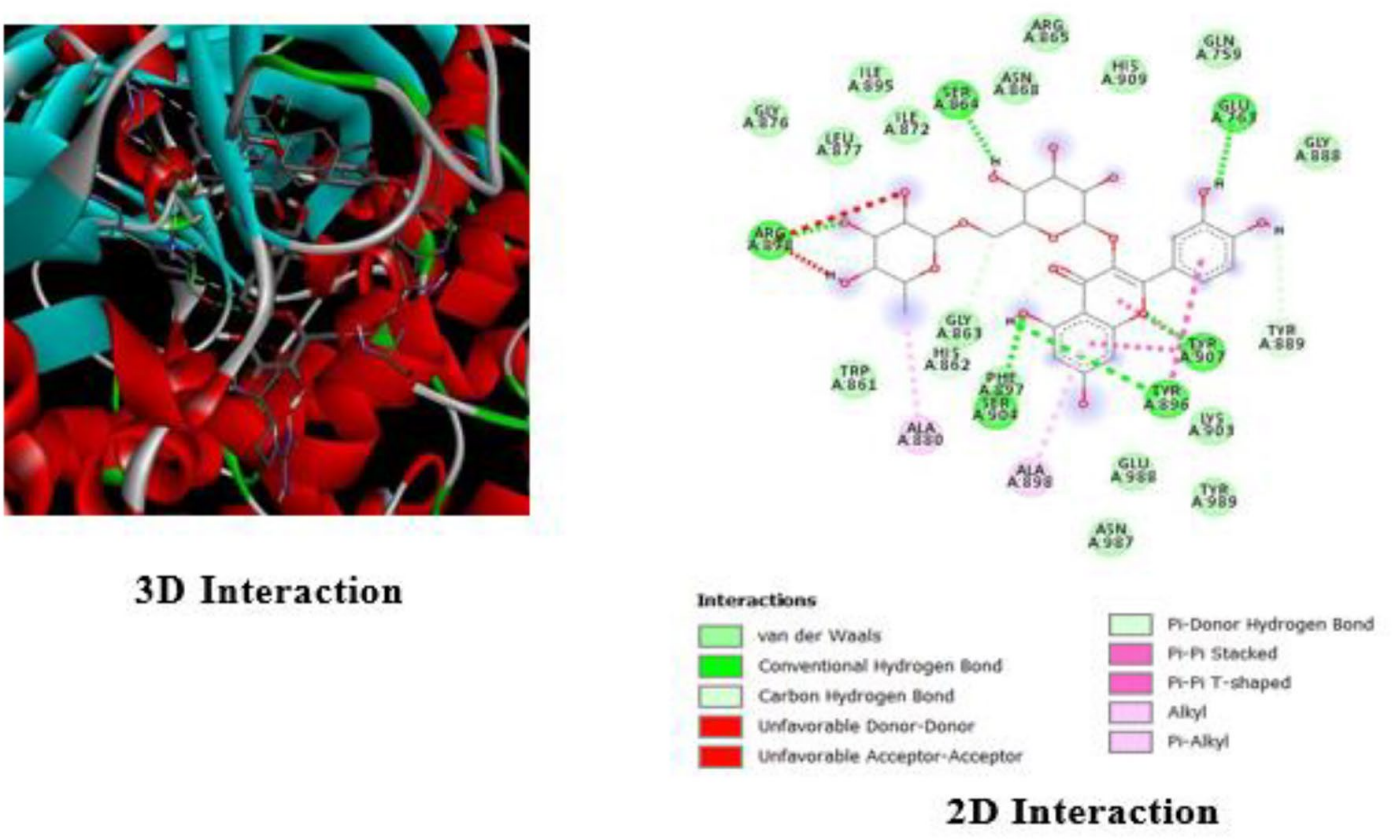
3D and 2D interaction of Rutin with PARP-1.

We employed Discovery Studio to visualize and analyze the interaction between Rutin and the PARP-1 receptor (Figs. 1 and 2). The investigation yielded valuable information regarding the binding of Rutin to the active site of PARP-1, elucidating the characteristics of their molecular interactions. Rutin forms six hydrogen bonds with the amino acid residues SER864, ARG878, SER904, TYR896, TYR907, and GLU763. These interactions are significant for the binding affinity of the compound. The molecule's binding stability is enhanced by the presence of additional stabilizing hydrophobic interactions involving ALA880 and ALA898. The 3D model depicts the spatial arrangement of Rutin within the active site of PARP-1. Dotted lines are used to represent the dynamic interactions between Rutin and certain amino acid residues.

**Figure 3:**
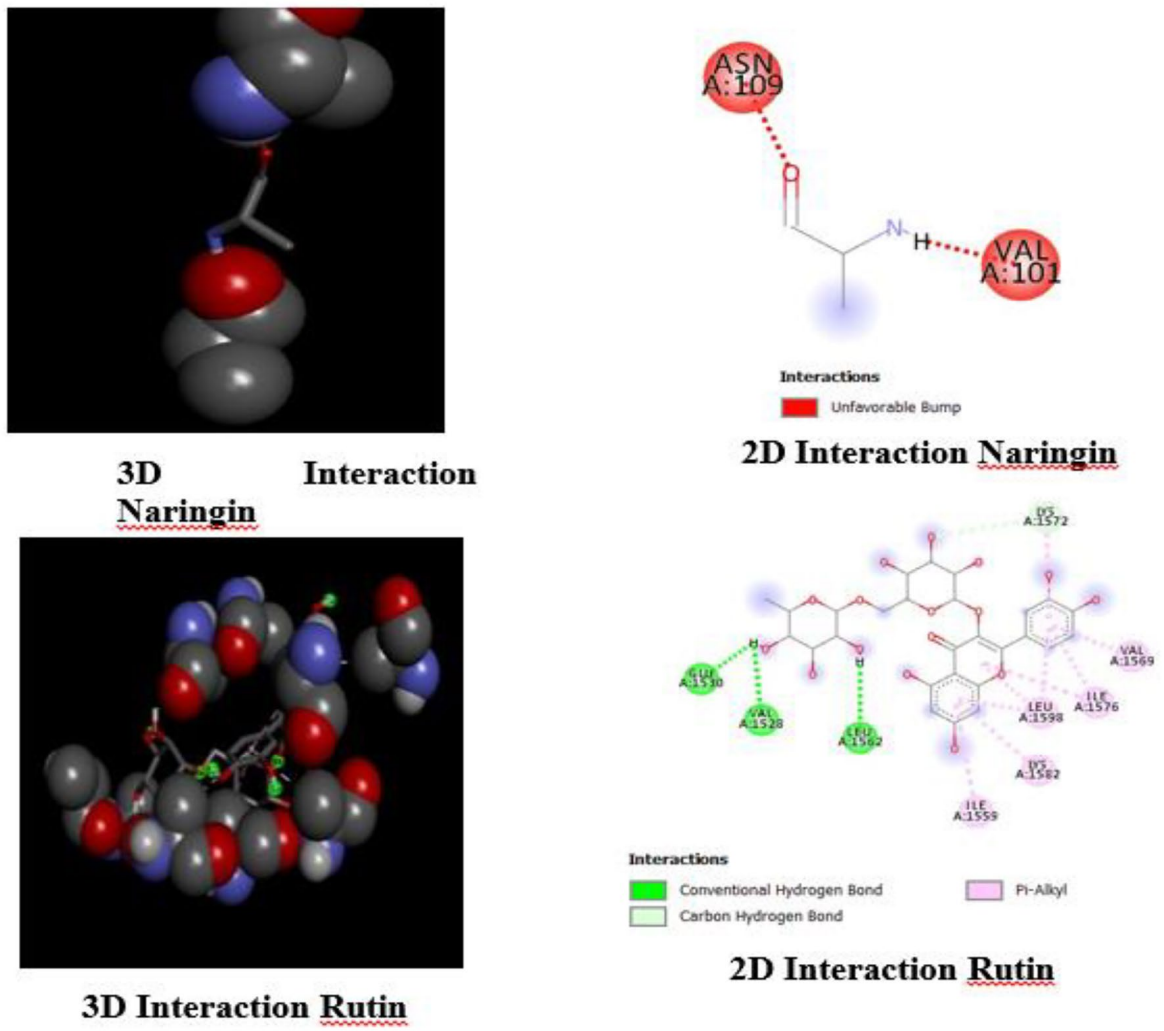
3D and 2D interaction of Naringin and Rutin with ATM.

We analyzed the binding interactions between Naringin and Rutin with the ATM protein using Discovery Studio. Our focus was on examining how these molecules interface with the active site of the ATM protein. The research identified a detrimental clash between Naringin and ATM at amino acid residues ASN109 and VAL101, which are shown in red. This clash suggests the possibility of spatial problems inside the binding region. Rutin has strong affinity, establishing three hydrogen bonds with GLU1530, VAL1528, and LEU1562 (shown in green), as well as five Pi-Alkyl connections with ILE1559, LYS1582, ILE1576, and VAL1569 (indicated in pink). Additionally, it forms a carbon-hydrogen bond with LYS1572. The interactions are shown in three dimensions using green circles and dotted lines, illustrating Rutin's successful alignment inside the ATM active site. This research offers a comparative perspective on the interactions between Naringin and Rutin with ATM. Figure 3 gives a detailed depiction of these interactions.

**Figure 4:**
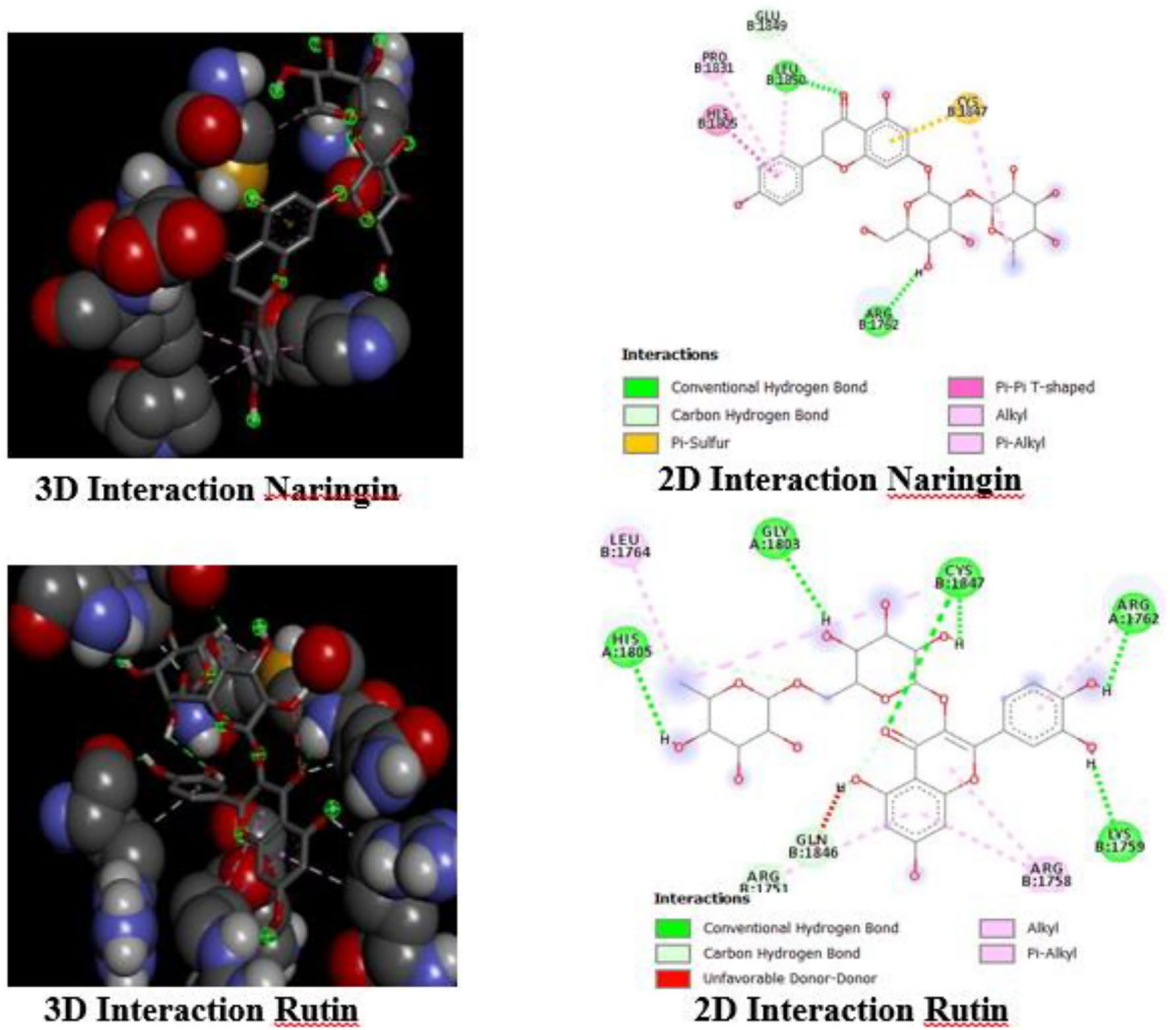
3D and 2D interaction of Naringin and Rutin with ATR.

Figure 4 illustrates the process of how Naringin and Rutin attach to the ATR protein. This provides a better understanding of how they interact at a molecular level within the active site of ATR. The analysis was conducted using Discovery Studio. Naringin establishes two hydrogen bonds with LEU1850 and ARG1762 (highlighted in green), suggesting particular sites of attachment that are likely to enhance its binding effectiveness. The 3D image provides additional information about these interactions through the use of green circles and dotted lines.

Rutin, on the other hand, demonstrates an intricate interaction pattern by forming five hydrogen bonds (with HIS1805, GLY1803, CYS1847, ARG1762, and LYS1759, indicated in green) as well as additional alkyl and Pi-Alkyl bonds (with ARG1758 and LEU1764, shown in pink). Additionally, it forms a carbon-hydrogen bond with GLN1846 and ARG1751. The interactions are depicted in both two-dimensional (2D) and three-dimensional (3D) formats, emphasizing Rutin's significant involvement with the ATR active site.

These observations provide a detailed account of the distinct interactions between Naringin and Rutin with ATR, indicating diverse ways they affect the protein's activity.

**Figure 5:**
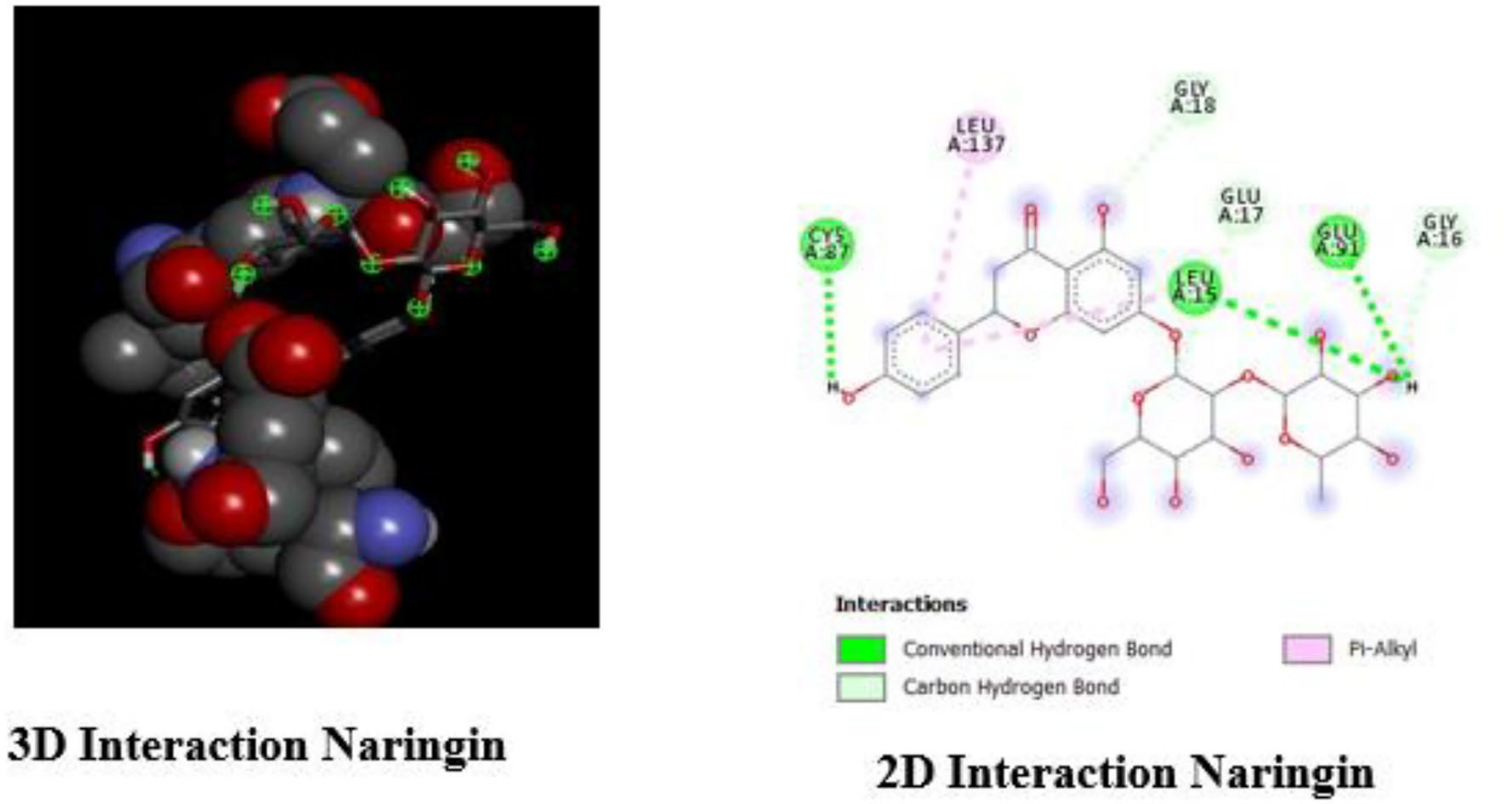
3D and 2D interaction of Naringin with CHK1.

Figure 5 illustrates the three-dimensional (3D) and two-dimensional (2D) interactions between Naringin and the CHK1 protein. It provides a vivid depiction of how Naringin binds to the active site of CHK1. By utilizing Discovery Studio, we discovered the following interactions: Naringin forms hydrogen bonds with CYS87, LEU15, and GLU91, which are shown in green. These connections indicate particular areas of attachment that enhance its affinity for CHK1. Additionally, Naringin's binding process includes a Pi-Alkyl interaction with LEU137. Naringin forms carbon-hydrogen bonds with GLY18, GLU17, and GLY16, which further enhances its interaction with CHK1 and increases stability. The intricate 2D and 3D visualizations emphasize Naringin's extensive interaction with CHK1, indicating a potentially substantial influence on CHK1's role in the DDR pathway.

**Figure 6:**
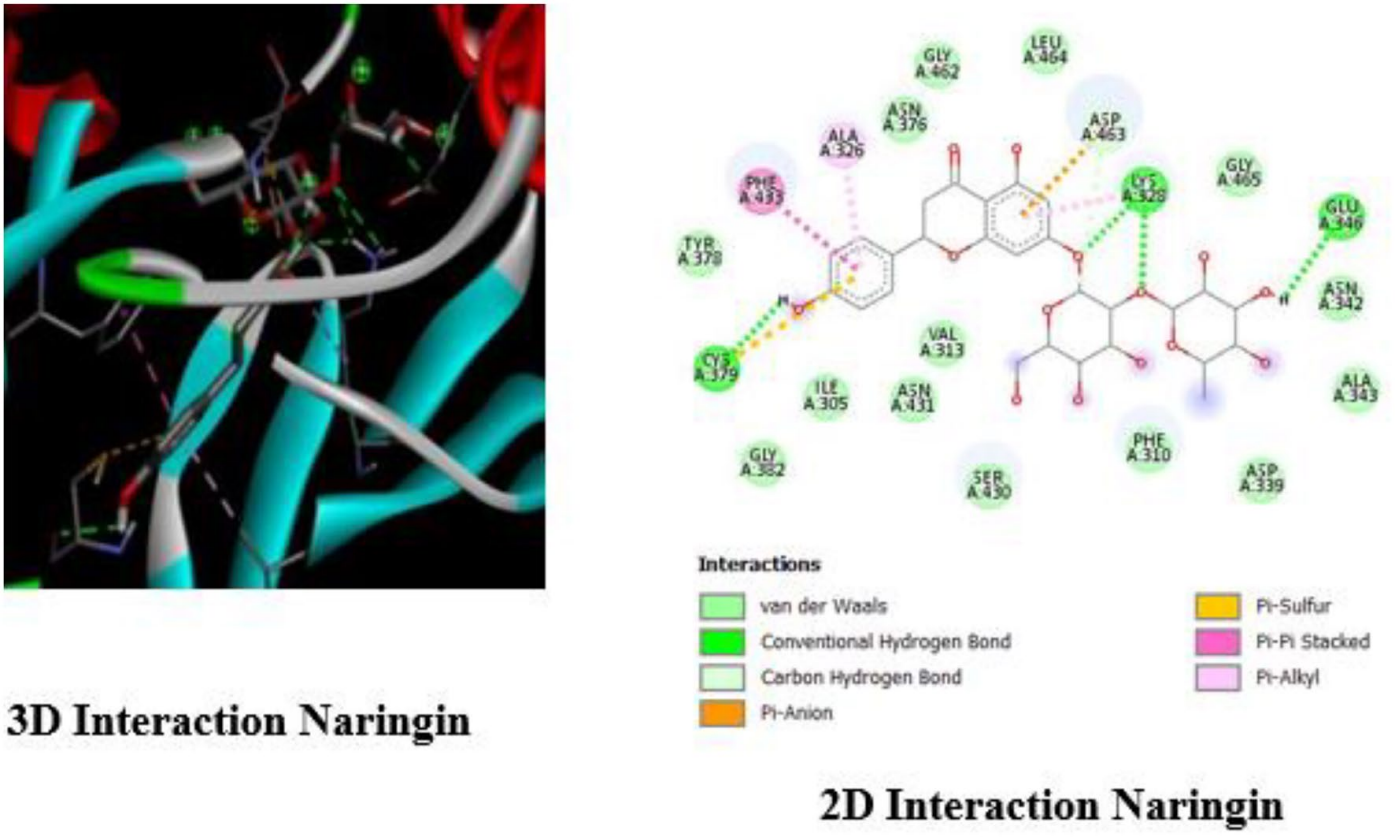
3D and 2D interaction of Naringin with WEE1.

Figure 6 illustrates the 3D and 2D interactions between Naringin and WEE1, demonstrating how Naringin interacts with the active site of WEE1. This research, performed using Discovery Studio, elucidates the characteristics of the chemical bonds established, emphasizing the particularity of Naringin's interaction with the WEE1 protein. Naringin establishes three hydrogen bonds with CYS379, LYS328, and GLU346, which are essential for its strong binding ability. These interactions are illustrated in green. The interaction also involves Pi-Alkyl and Pi-Pi stacking bonds with ALA326 and PHE433, suggesting a complex and varied interaction with the protein. The presence of a carbon-hydrogen bond at ASP463 enhances the stability and specificity of Naringin's binding to WEE1, hence increasing the level of interaction. The information obtained from the 2D and 3D visualizations highlights the possible impact of Naringin on the function of WEE1, providing vital understanding of how it works within the DDR pathway.

### ADMET profiles of Naringin and Rutin

**Figure 7:**
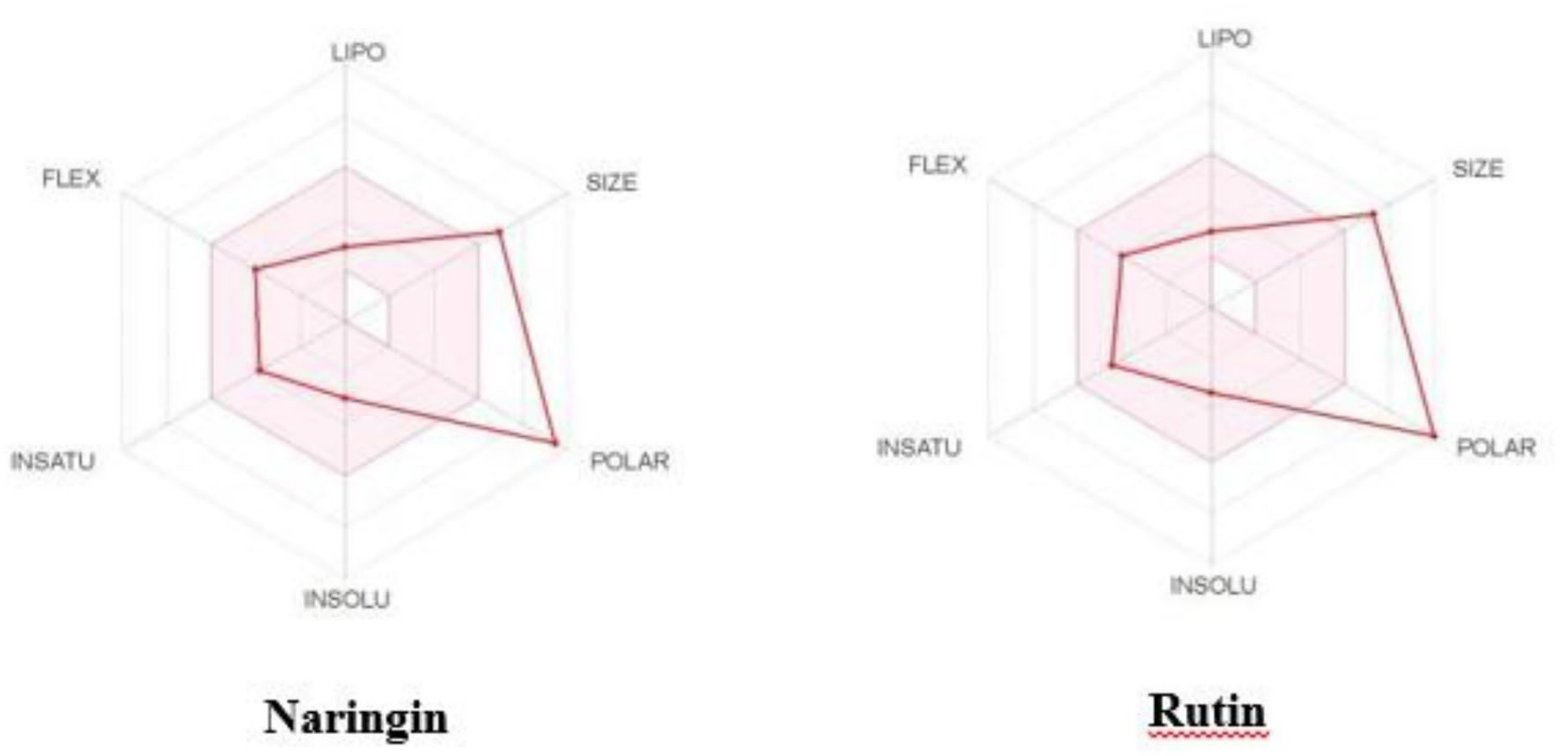
Naringin and Rutin SWISS profile.

We employed SWISS ADMET and PkCSM ADMET methods to assess the ADMET (Absorption, Distribution, Metabolism, Excretion, and Toxicity) characteristics and drug-likeness of Naringin and Rutin in our study. The computational predictions offer a thorough analysis of the compounds' physical and chemical characteristics, how they are processed in the body, and their qualities in the field of medicinal chemistry. SWISS ADMET provided information on the physicochemical characteristics, lipophilicity, water solubility, and pharmacokinetics of Naringin and Rutin, indicating their potential as medication candidates. The comprehensive statistics are contained in Figure 7 and Table 3.

**Table 3:** ADMET SWISS profile of Naringin and Rutin.

Sr No	Properties	Naringin	Rutin
Physicochemical properties			
1	Formula	C27H32O14	C27H30O16
2	Molecular weight	580.53 g/mol	610.52 g/mol
3	Num. heavy atoms	41	43
4	Num. arom. heavy atoms	12	16
5	Fraction Csp3	0.52	0.44
6	Num. rotatable bonds	6	6
7	Num. H-bond acceptors	14	16
8	Num. H-bond donors	8	10
9	Molar Refractivity	134.91	141.38
10	TPSA	225.06 Å^2^	269.43 Å^2^
Lipophilicity			
11	Log *P*_o/w_ (iLOGP)	2.38	2.43
12	Log *P*_o/w_ (XLOGP3)	− 0.44	− 0.33
13	Log *P*_o/w_ (WLOGP)	− 1.49	− 1.69
14	Log *P*_o/w_ (MLOGP)	− 2.77	− 3.89
15	Log *P*_o/w_ (SILICOS-IT)	− 1.64	− 2.11
16	Consensus Log *P*_o/w_	− 0.79	− 1.12
Water solubility			
17	Log *S* (ESOL)	− 2.98	− 3.30
18	Solubility	6.04e − 01 mg/ml ; 1.04e − 03 mol/l	3.08e − 01 mg/ml ; 5.05e− 04 mol/l
19	Class	Soluble	Soluble
20	Log *S* (Ali)	− 3.82	− 4.87
21	Solubility	8.77e − 02 mg/ml ; 1.51e − 04 mol/l	8.30e − 03 mg/ml ; 1.36e − 05 mol/l
22	Class	Soluble	Moderately soluble
23	Log *S* (SILICOS-IT)	− 0.49	− 0.29
24	Solubility	1.87e + 02 mg/ml ; 3.21e − 01 mol/l	3.15e + 02 mg/ml ; 5.15e − 01 mol/l
25	Class	Soluble	Soluble
Pharmacokinetics			
26	GI absorption	Low	Low
27	BBB permeant	No	No
28	P-gp substrate	Yes	Yes
29	CYP1A2 inhibitor	No	No
30	CYP2C19 inhibitor	No	No
31	CYP2C9 inhibitor	No	No
32	CYP2D6 inhibitor	No	No
33	CYP3A4 inhibitor	No	No
34	Log *K*_p_ (skin permeation)	− 10.15 cm/s	− 10.26 cm/s
Druglikeness			
35	Lipinski	No; 3 violations: MW > 500, NorO > 10, NHorOH > 5	No; 3 violations: MW > 500, NorO > 10, NHorOH > 5
36	Ghose	No; 4 violations: MW > 480, WLOGP < -0.4, MR > 130, #atoms > 70	No; 4 violations: MW > 480, WLOGP < -0.4, MR > 130, #atoms > 70
37	Veber	No; 1 violation: TPSA > 140	No; 1 violation: TPSA > 140
38	Egan	No; 1 violation: TPSA > 131.6	No; 1 violation: TPSA > 131.6
39	Muegge	No; 3 violations: TPSA > 150, H- acc > 10, H-don > 5	No; 4 violations: MW > 600, TPSA > 150, H- acc > 10, H-don > 5
40	Bioavailability Score	0.17	0.17
Medicinal chemistry			
41	PAINS	0 alert	1 alert: catechol_A
42	Brenk	0 alert	1 alert: catechol
43	Leadlikeness	No; 1 violation: MW > 350	No; 1 violation: MW > 350
44	Synthetic accessibility	6.16	6.52

We utilized PkCSM ADMET to validate the ADMET profiles of Naringin and Rutin, enhancing our understanding of their pharmacokinetic properties. This analysis covered aspects of absorption, distribution, metabolism, excretion, and toxicity, crucial for evaluating the compounds' suitability as drug candidates (Table 4).

**Table 4:** ADMET PkCSM profile of Naringin and Rutin.

Sr No	Properties	Predicted Value of Naringin	Predicted Value of Rutin	Unit
Absorption				
1	Water solubility	− 2.919	− 2.892	Numeric (log mol/L)
2	Caco2 permeability	− 0.658	− 0.949	Numeric (log Papp in 10^6^ cm/s)
3	Intestinal absorption (human)	25.796	23.446	Numeric (% Absorbed)
4	Skin Permeability	− 2.735	− 2.735	Numeric (log Kp)
5	P-glycoprotein substrate	Yes	Yes	Categorical (Yes/No)
6	P-glycoprotein I inhibitor	No	No	Categorical (Yes/No)
7	P-glycoprotein II inhibitor	No	No	Categorical (Yes/No)
Distribution				
8	VDss (human)	0.619	1.663	Numeric (log L/kg)
9	Fraction unbound (human)	0.159	0.187	Numeric (Fu)
10	BBB permeability	− 1.6	− 1.899	Numeric (log BB)
11	CNS permeability	− 4.773	− 5.178	Numeric (log PS)
Metabolism				
12	CYP2D6 substrate	No	No	Categorical (Yes/No)
13	CYP3A4 substrate	No	No	Categorical (Yes/No)
14	CYP1A2 inhibitor	No	No	Categorical (Yes/No)
15	CYP2C19 inhibitor	No	No	Categorical (Yes/No)
16	CYP2C9 inhibitor	No	No	Categorical (Yes/No)
17	CYP2D6 inhibitor	No	No	Categorical (Yes/No)
18	CYP3A4 inhibitor	No	No	Categorical (Yes/No)
Excretion				
19	Total clearance	0.318	− 0.369	Numeric (log ml/min/kg)
20	Renal OCT2 substrate	No	No	Categorical (Yes/No)
Toxicity				
21	AMES toxicity	No	No	Categorical (Yes/No)
22	Max. tolerated dose (human)	0.43	0.452	Numeric (log mg/kg/day)
23	hERG I inhibitor	No	No	Categorical (Yes/No)
24	hERG II inhibitor	Yes	Yes	Categorical (Yes/No)
25	Oral Rat Acute Toxicity (LD50)	2.495	2.491	Numeric (mol/kg)
26	Oral Rat Chronic Toxicity (LOAEL)	4.202	3.673	Numeric (log mg/kg_bw/day)
27	Hepatotoxicity	No	No	Categorical (Yes/No)
28	Skin Sensitisation	No	No	Categorical (Yes/No)
29	*T.Pyriformis* toxicity	0.285	0.285	Numeric (log ug/L)
30	Minnow toxicity	6.042	7.677	Numeric (log mM)

## MTT assay

### MTT assay of Rutin on 2DD Normal Fibroblasts and MDA-MB-231 Breast Cancer Cells

**Figure 8:**
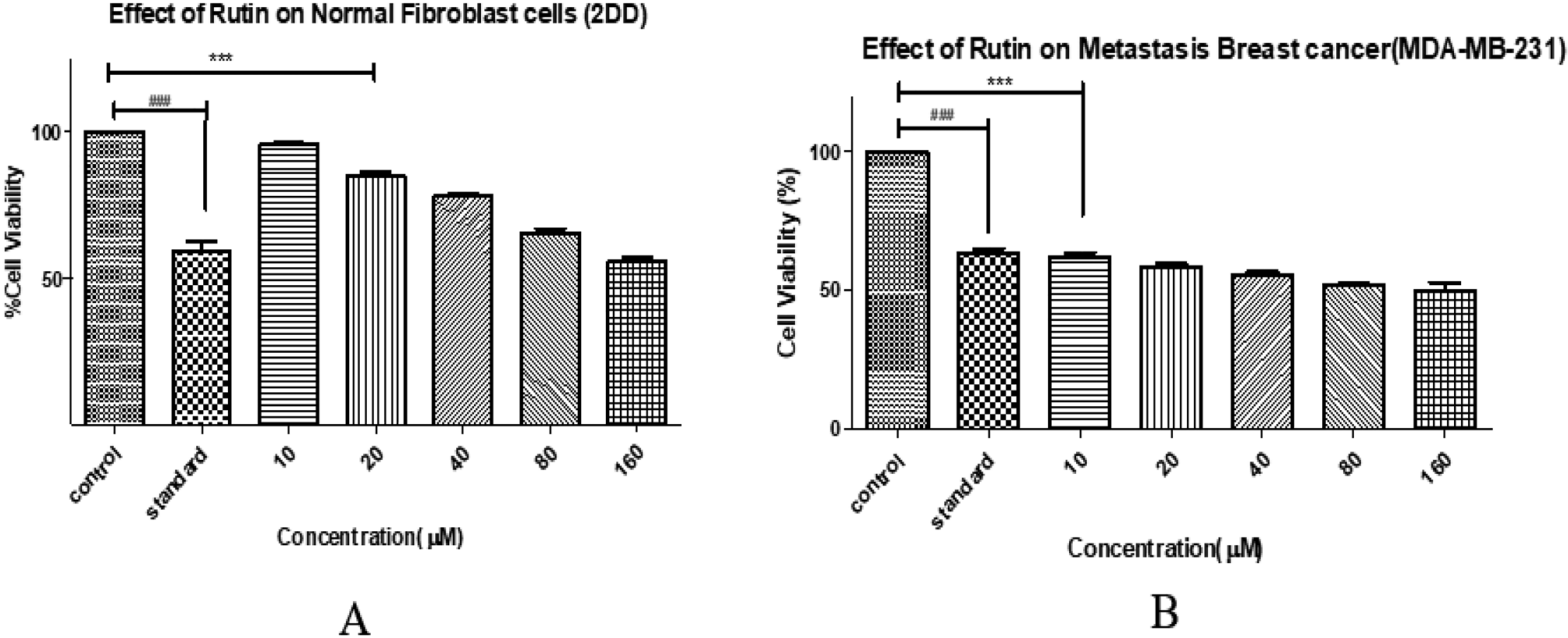
Comparative Cytotoxicity of Rutin on 2DD Normal Skin Fibroblast Cells (**A**) and MDA-MB-231 Breast Cancer Cells (**B**) as Assessed by MTT Assay. The data, represented as means ± Standard Deviation, was subjected to statistical analysis using ANOVA, followed by a post-hoc Tukey test to determine the significance levels (***P < 0.0001).

The cytotoxicity of Rutin was assessed using MTT assays at various doses (0–160 µM) on 2DD normal skin fibroblast cells and MDA-MB-231 breast cancer cells in this study. The experiments, performed in triplicate, were designed to ascertain the specific toxicity of Rutin. The data, represented as means ± standard deviation, were subjected to statistical analysis using ANOVA, followed by a post-hoc Tukey test to determine the significance levels (***P < 0.0001) (Figure 8).

This study aimed to evaluate the cytotoxic effects of Rutin using MTT assays on 2DD normal skin fibroblast cells and MDA-MB-231 breast cancer cells. Rutin concentrations ranging from 0 to 160 µM were tested. The assay was performed in triplicate to confirm the reliability of the data. The investigation showed that cell viability in normal 2DD fibroblast cells maintained between 50–95% for all tested Rutin dosages, suggesting less cytotoxic effect compared to the untreated control group.

It is worth mentioning that Anastrozole, used as a standard for comparison, showed a considerable level of cell-killing ability compared to both the control group and different doses of Rutin. This highlights the effectiveness of Anastrozole as a reference point in our experiments.

On the other hand, there was a clear and direct relationship between the amount of Rutin and the level of cell death in MDA-MB-231 breast cancer cells. As the concentration of Rutin increased, cytotoxicity also increased. This increasing toxicity demonstrates Rutin's promise as an anticancer agent, specifically targeting breast cancer cells.

Based on the gathered data, we determined that the IC50 value of Rutin for MDA-MB-231 cells is 45 µM. This figure shows the concentration at which Rutin is capable of inhibiting 50% of cell viability, acting as a critical indicator of its cytotoxic effectiveness against cancer cells while demonstrating its protective impact on normal cells.

### MTT assay of Naringin on 2DD Normal Fibroblasts and MDA-MB-231 Breast Cancer Cells

**Figure 9:**
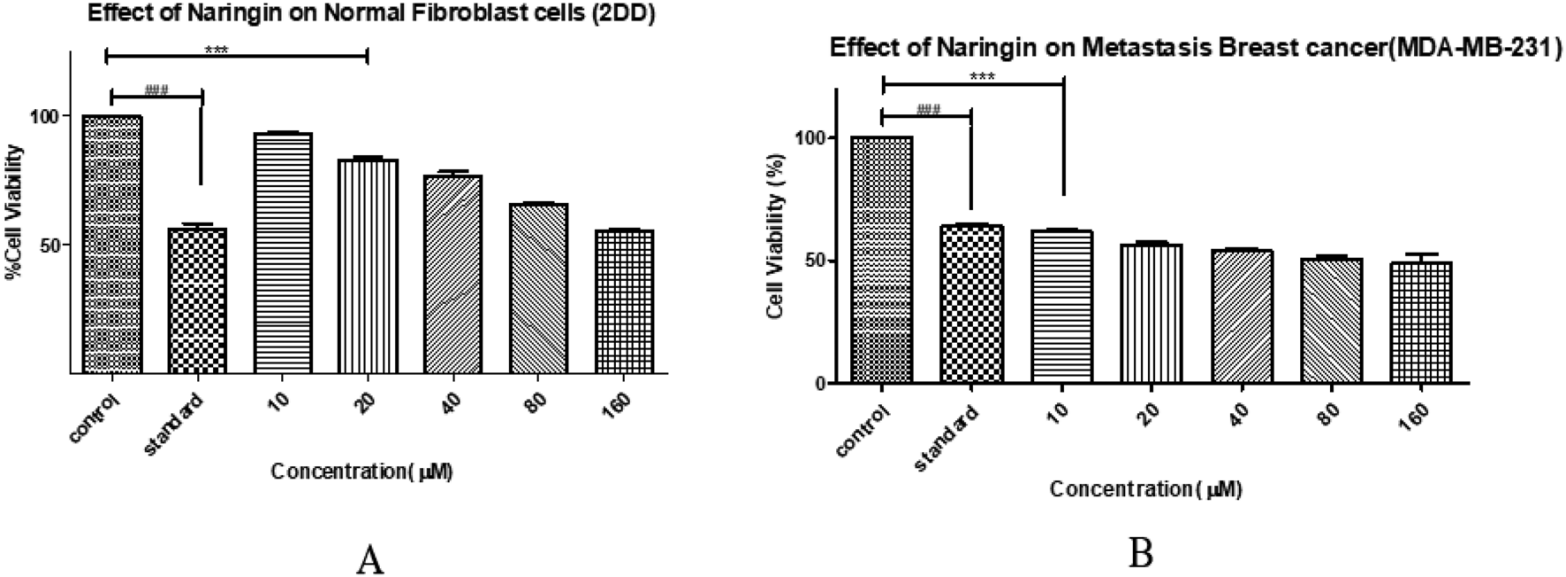
MTT Assay Analysis of Naringin's Cytotoxic Effects on 2DD Normal Skin Fibroblast and MDA-MB-231 Breast Cancer Cells. The data, represented as means ± Standard Deviation, was subjected to statistical analysis using ANOVA, followed by a post-hoc Tukey test to determine the significance levels (***P < 0.0001).

MTT assays were conducted to examine the cytotoxic effects of Naringin on 2DD normal skin fibroblast cells and MDA-MB-231 breast cancer cells. Tested at a concentration range of 0–160 µM. Figure 9 depicts the findings of MTT tests that assessed the cytotoxic effects of the Naringin compound on 2DD normal skin fibroblast cells and MDA-MB-231 breast cancer cells at various doses (ranging from 0 to 160 µM). The experiments, carried out to determine the specific cytotoxic effects of Naringin, were repeated three times to guarantee precision and consistency. The data acquired, which represents cell viability, is presented as the mean value plus or minus the standard deviation. Figure 9C illustrates the effect of different concentrations of Naringin on the survival of 2DD normal skin fibroblast cells. Figure 9D, on the other hand, examines the toxic reaction of MDA-MB-231 breast cancer cells to changing concentrations of Naringin.

The experiment was conducted in triplicate. Analysis of the data showed that the viability of normal 2DD fibroblast cells ranged from 50 to 95% at different dosages of Naringin. This suggests that these cells were not experience minor toxicity compared to the control group. Anastrozole, used as the benchmark, exhibited a notable disparity in cytotoxicity when compared to both the untreated control and various doses of Naringin.

On the other hand, as the concentration of Naringin increased, there was an increased toxicity to MDA-MB-231 breast cancer cells, indicating its capacity to specifically target and harm cancer cells. The experiments showed that Naringin did not have any harmful effects on normal cells within the tested dose range. However, it exhibits significant toxicity against breast cancer cells, as shown in Fig. 9. Based on the gathered data, we determined that the IC50 value of Naringin for MDA-MB-231 cells is 16 µM. This figure shows the concentration at which Naringin is capable of inhibiting 50% of cell viability, acting as a critical indicator of its cytotoxic effectiveness against cancer cells while demonstrating its protective impact on normal cells.

### DPPH assay

The DPPH assay was performed to evaluate the ability of Rutin and Naringin to scavenge free radicals. The experiment was done in triplicates, using doses similar to those used in the MTT assay. Quercetin, known for its strong antioxidant capabilities, was used as the reference standard. The antioxidant activities were quantified and expressed as means ± Standard Deviation. The observed differences were evaluated using ANOVA followed by a post-hoc Tukey test. This analysis identified significant variances (***P < 0.0001) in the scavenging efficacy across different concentrations of molecules.

**Figure 10:**
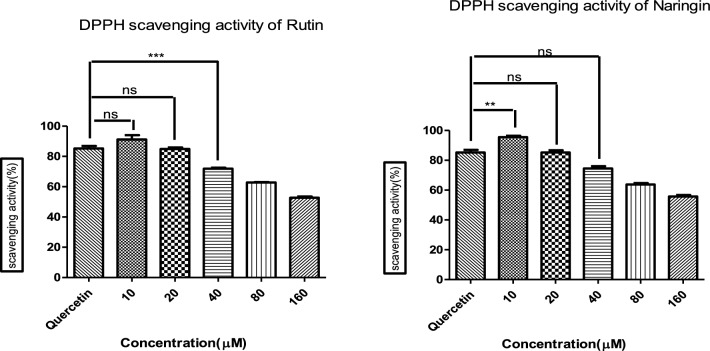
Evaluation of Free Radical Scavenging Activity by Rutin and Naringin Using DPPH Assay. The data, represented as means ± Standard Deviation, was subjected to statistical analysis using ANOVA, followed by a post-hoc Tukey test to determine the significance levels (***P < 0.0001, **P < 0.001).

Figure 10 depicts the results of the DPPH assay. Statistical analysis was done using ANOVA followed by the Tukey test, which revealed notable variations in activity. The p-value, which was less than 0.0001, indicates a strong antioxidant capability of the substances being examined.

The results of our study show that both Rutin and Naringin have strong antioxidant properties at lower doses, however, these properties decrease significantly, as the concentrations grow. Unlike Quercetin, which showed a constant degree of antioxidant activity at both 10 and 20 µM concentrations, the antioxidant activity of this substance varies depending on its concentration. After analyzing these data, a concentration of 10 µM was chosen to conduct a more in-depth investigation into the scavenging abilities of Rutin and Naringin, as seen in Fig. 10.

### Free radical assays

Our study on the antioxidant properties of Rutin and Naringin, using free radical assays, demonstrated a complex activity profile against many types of radicals, such as hydroxyl (OH), peroxynitrite (ONOO), superoxide (O2 −), and nitric oxide (NO). Remarkably, Naringin demonstrated a strong ability to remove harmful radicals, exceeding 60% for all tested radicals. This discovery strongly confirms its powerful antioxidant characteristics. The results obtained from experiments done at a consistent concentration of 10 µM were thoroughly confirmed using statistical analysis. This involved applying ANOVA followed by the Tukey test, which demonstrated the significance of these findings with a *P* value of less than 0.0001.

**Figure 11:**
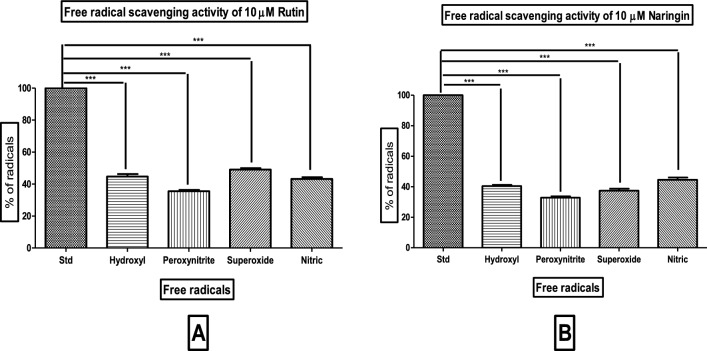
Comparative free radical scavenging activity of Rutin and Naringin Against Various Free Radicals. The data, represented as means ± Standard Deviation, was subjected to statistical analysis using ANOVA, followed by a post-hoc Tukey test to determine the significance levels (***P < 0.0001).

Figure 11 depicts the results of the free radical scavenging experiment, which assesses the efficacy of Rutin (Panel A) and Naringin (Panel B) in counteracting hydroxyl radical (OH), peroxynitrite (ONOO), superoxide (O2 −), and nitric oxide (NO) radicals at a consistent concentration of 10 µM. The compounds exhibited considerable antioxidant capacity against these radicals, as evidenced by the P value of less than 0.0001 obtained from ANOVA and the subsequent Tukey test.

## Single Cell gel electrophoresis (Comet Assay)

### Rutin and Naringin effect on DNA base and DNA strand break in Normal Quiescent Fibroblast cells and MDA-MB-231 Breast cancer cells

Our work employed comet assays to evaluate the oxidative damage impact of Rutin and Naringin on DNA base and strand breaks in both normal and cancer cells, specifically at a concentration of 10 µM. The inclusion of quiescent fibroblast cells in comet tests was crucial due to their established sensitivity to oxidative stress and their dynamic metabolic profile, which is advantageous for investigating DNA damage. Their distinctive attributes greatly enhance the sensitivity of comet tests, rendering them ideal for this research^38,39^.

**Figure 12:**
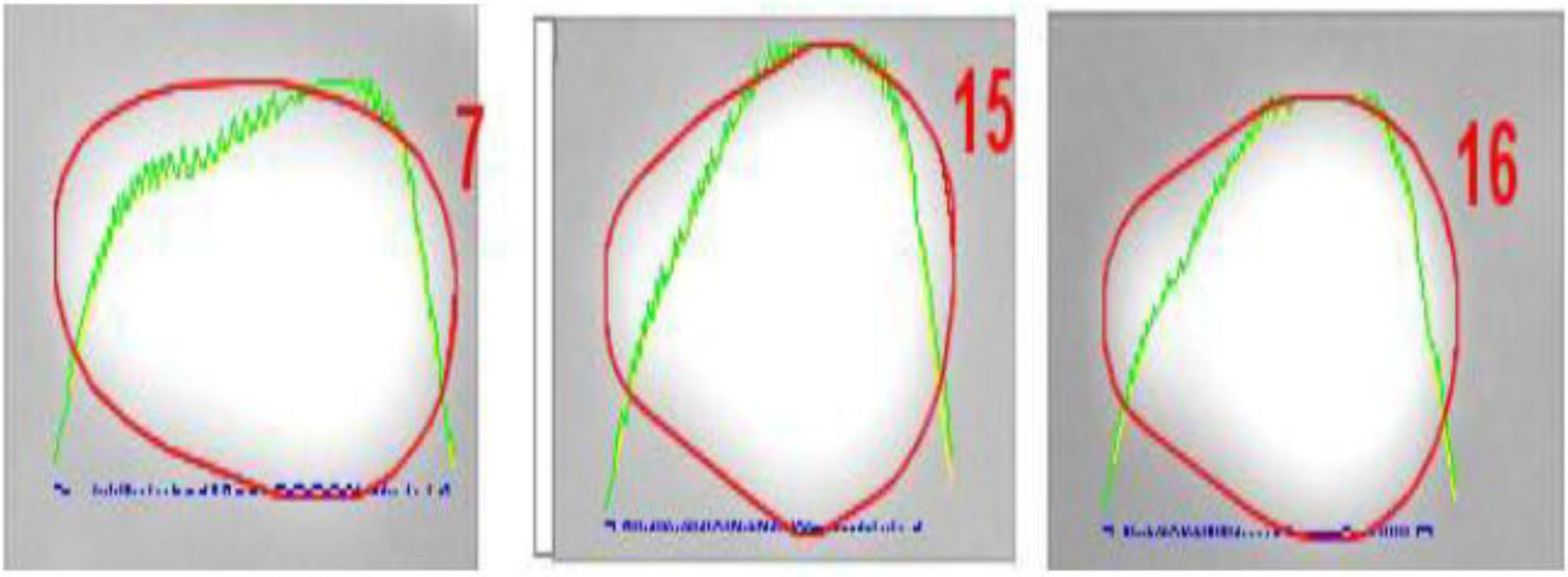
Representative Images of quiescent fibroblast cells from untreated slides analyzed by OpenComet plugin in ImageJ. Cell without COMET.

**Figure 13:**
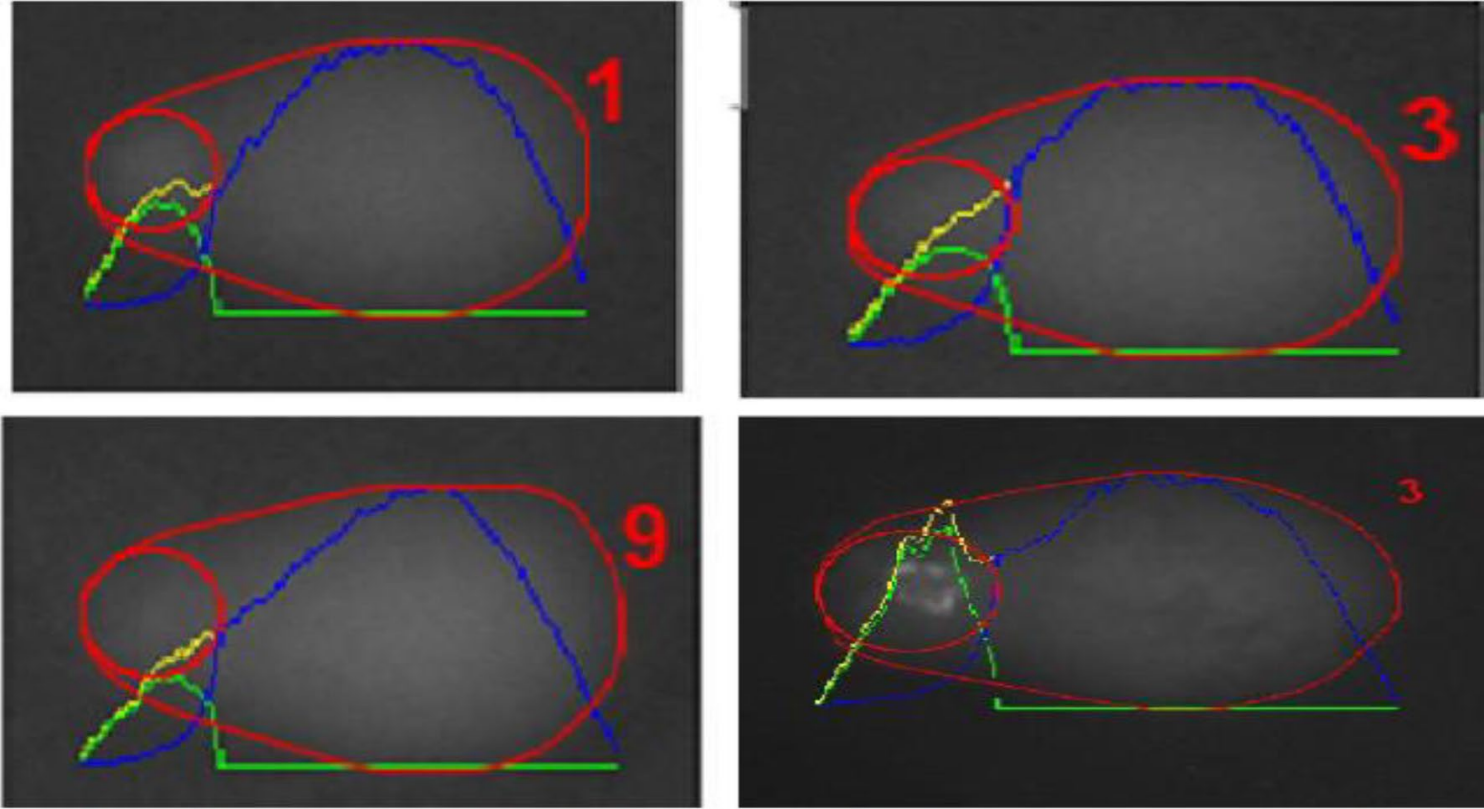
Representative Images of quiescent fibroblast cells from treated slides analysed by OpenComet plugin in ImageJ. Cell with COMET.

**Figure 14:**
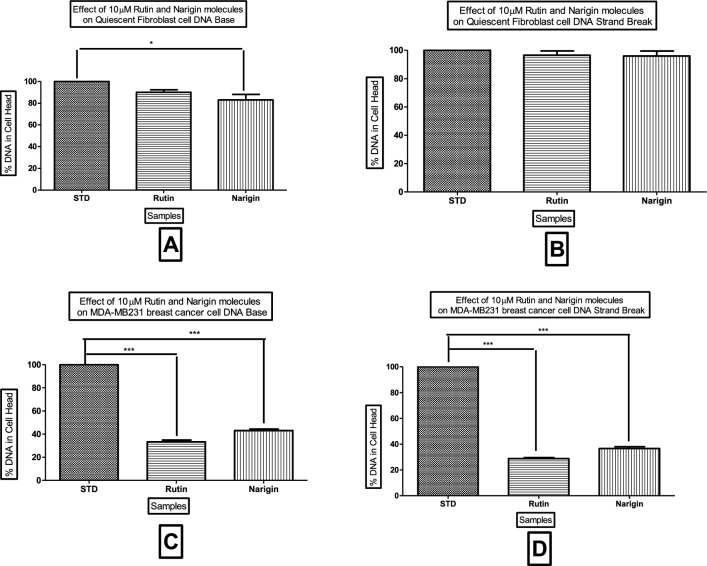
Assessment of DNA Damage Induced by Naringin and Rutin in Quiescent Fibroblast and Breast Cancer Cells Using Comet Assay treated with 10 µM of Naringin and Rutin. The data, represented as means ± Standard Deviation, was subjected to statistical analysis using ANOVA, followed by a post-hoc Tukey test to determine the significance levels (***P < 0.0001).

This investigation entailed the examination of DNA within the nuclei of 200 treated cells, with the results being expressed as a percentage of DNA damage in the nucleus. A comparative examination was conducted between cells that were not treated (STD) and cells that were treated with Rutin and Naringin. Interestingly, Rutin and Naringin did not exhibit any genotoxic effects in normal cells. However, at the concentration of 10 µM, a substantial increase in DNA damage was detected in breast cancer cells. The results showed a 60–70% rise in DNA damage, as seen in Figs. 12, 13, and 14.

A high percentage of DNA in the cell head is a sign of less or no DNA damage in the cell. In Figure A, we can see the effect of the sample on DNA bases in quiescent fibroblast cells (QFC). Figure B depicts DNA strand breaks in quiescent fibroblast cells (QFC). Figure C shows DNA base damage in MDA-MB-231 breast cancer cells. Figure D exhibits DNA strand breaks in MDA-MB-231 breast cancer cells. Each experiment was conducted at a concentration of 10 µM. To guarantee reproducibility, each experiment was performed in triplicate. The data represented specifically examines the comparative study of the different forms of DNA damage caused by Naringin and Rutin in both cell models.

## Discussion

Through our analysis using PyRx and Discovery Studio, we have successfully elucidated the intricate molecular interactions between Naringin and Rutin with crucial proteins in the DNA damage response (DDR) pathway, which play significant roles in DNA repair. This has allowed us to uncover the distinctiveness and variety of their binding affinities. The comprehensive research reveals that Naringin extensively interacts with proteins including ATM, ATR, CHK1, and WEE1, whereas Rutin demonstrates a strong affinity towards PARP-1, ATM, and ATR.

This indicates that these natural compounds may have a regulatory effect on the DDR process, which could be relevant for therapeutic treatments, especially in cancer where DDR regulation is essential. The analysis of the binding patterns, such as hydrogen bonds and Pi interactions, highlights the potential of Naringin and Rutin as natural regulators of DDR protein activity. These interactions suggest that these compounds could influence how cells respond to DNA damage, potentially leading to novel approaches in cancer treatment. This is particularly relevant for strategies aimed at sensitizing cancer cells to DNA-damaging drugs or overcoming drug resistance mechanisms.

The ADMET and drug-likeness tests performed on Naringin and Rutin, utilizing SWISS ADMET and PkCSM ADMET tools, reveal the delicate balance between their favorable pharmacological characteristics and the challenges they face in the process of drug development. These compounds demonstrate advantageous solubility and minimal inhibition of cytochrome P450, indicating their potential as candidates for therapeutic development.

However, the physicochemical characteristics of these substances, such as their elevated molecular weight and the number of hydrogen bond donors and acceptors, exceed the optimal limits established by Lipinski's "Rule of Five." This discrepancy raises concerns regarding their capacity for oral absorption and their ability to pass through cell membranes. Additionally, their classification as P-glycoprotein substrates and the predictions of limited gastrointestinal absorption emphasize the need for optimization to ensure efficient delivery and therapeutic efficacy.

The MTT assay provides a clear understanding of the cytotoxic effects of Rutin and Naringin, revealing their potential as effective cancer treatments. The concentration-dependent cytotoxicity observed in these experiments highlights a crucial characteristic of these natural compounds: their ability to specifically target cancer cells while having minimal impact on normal cells. The selectivity of the compounds is evident in their capacity to maintain cell viability within the range of 50–95% in normal cells, even when exposed to concentrations ranging from 0–160 µM. In contrast, increasing concentrations of the compounds have been found to cause greater toxicity in MDA-MB-231 breast cancer cells. These findings suggest that these chemicals have a wider range of effectiveness in treating cancer, indicating their potential for safer treatment methods.

The pronounced cytotoxicity observed in cancer cells, in contrast to the minimal effect on normal cells, is consistent with previous research that highlights the specific targeting of plant-derived chemicals against malignant tissues. The research conducted by Alshwyeh et al.^40^ and Nahar et al.^41^ supports the idea that natural chemicals can specifically target cancer cells while sparing normal cells. This characteristic reduces the potential harm to healthy cells, making these compounds promising candidates for anticancer treatments. This is further supported by the comparison analysis with Anastrozole, highlighting the effectiveness of Rutin and Naringin in a therapeutic environment.

Furthermore, the fact that no harmful effects were observed in healthy cells even at doses as high as 160 µM for both Rutin and Naringin sets an important standard for safety. This implies that these chemicals have the potential to be used in therapeutic applications without causing harm to healthy tissue. This is supported by the ability of Rutin to induce apoptosis in gastric cancer cells, as demonstrated by Li et al.^42^. The features of Rutin and Naringin highlight their usefulness not only as anticancer agents but also as compounds that have a positive safety profile^43^.

The distinct cytotoxic effects of Rutin and Naringin on malignant and non-cancerous cell lines highlight the need for further research to uncover the underlying mechanisms of action. Gaining a comprehensive understanding of these processes is crucial for the advancement of innovative cancer treatments that leverage the specific ability of natural substances to target cancer cells, intending to develop techniques that are both highly effective and free from the harmful side effects commonly associated with traditional chemotherapy. The findings related to Rutin and Naringin underscore the promise of natural compounds in enhancing cancer therapy towards more tailored and less harmful methods by preferentially targeting cancer cells.

The DPPH assay findings demonstrate a good antioxidant activity that varies with the quantity of Rutin and Naringin, in contrast to the steady activity reported for Quercetin. The observed trend indicates a subtle behavior of natural antioxidants, where Rutin and Naringin have a strong ability to neutralize free radicals at lower concentrations, but this ability decreases as the dosages increase. The phenomena discussed in our work align with findings in the literature, indicating an intricate relationship between concentration and the effectiveness of antioxidants in natural substances.

Research conducted by Ganash^43^ highlighted the antioxidant properties of flavonoids such as Rutin and Naringin. Rusmana et al. (2017) further corroborate this^44^, who observed that Quercetin has exceptional antioxidant activity. Limanto et al.^45^ expanded upon this comparison by highlighting the superior antioxidative and α-glucosidase inhibitory properties of Quercetin compared to Rutin. These findings emphasize the importance of dosage in maximizing the therapeutic advantages of flavonoids.

The choice to analyze Rutin and Naringin at a concentration of 10 µM is supported by their strong antioxidant properties at this level. This concentration provides a promising opportunity to thoroughly investigate their ability to remove harmful substances. The varying antioxidant responses of these substances, in contrast to the reliable effectiveness of Quercetin, underscore the crucial requirement for a detailed comprehension of dose-dependent effects in the advancement of antioxidant-focused therapies. Understanding this observation is essential for utilizing the healing properties of natural antioxidants effectively. It also emphasizes the need for focused scientific investigation to determine the optimal concentrations that provide the greatest effectiveness while minimizing any possible negative effects.

The found free radical scavenging capabilities in Rutin and Naringin demonstrate their potential as natural antioxidants, which is consistent with and builds upon previous studies in this field. The significant effectiveness of Naringin highlights its ability to reduce oxidative stress and shield against DNA damage^46–48^. These studies investigated Naringin's antioxidant properties and its protective role against DNA damage caused by radiation and H2O2, respectively.

On the other hand, the scavenging ability of Rutin against the measured radicals is considerably reduced. Although this is noteworthy, it indicates a complexity in its antioxidant action that requires additional investigation. This discovery is supported by previous studies in the literature. Yang^49^ and Peng et al.^50^ emphasized the efficiency of Rutin in scavenging hydroxyl and superoxide radicals. However, it is important to note that the effectiveness of Rutin may vary depending on the kind of radical. The heterogeneity mentioned highlights the complex nature of antioxidant processes and the impact of molecule structure on their ability to scavenge harmful substances.

The difference in scavenging behaviours between Rutin and Naringin, especially when compared to the reliable antioxidant effectiveness of well-known substances such as Quercetin, underscores the necessity for a more thorough examination of the interactions between these natural antioxidants and different types of radicals. Comprehending this is vital for effectively using their healing capabilities, particularly in situations where oxidative stress significantly contributes to the development of diseases.

This study not only confirms the antioxidant abilities of Rutin and Naringin, but also encourages further investigation into the specific ways in which their actions vary according to concentration. The investigation of the single-cell gel electrophoresis to understand the oxidative DNA damage profiles of these compounds, especially at lower concentrations, indicates potential uses for these substances in preventive and therapeutic approaches against DNA damage. This has implications for cancer prevention and the management of other conditions related to oxidative stress.

The work focused on the selective oxidative DNA damage of Rutin and Naringin in breast cancer cells, while not impacting normal quiescent fibroblast cells. This finding represents a significant advancement in the possible therapeutic use of natural chemicals. These findings are consistent with the need for a sophisticated strategy in cancer treatment, where it is equally important to minimize damage to healthy cells as it is to target cancer cells.

The use of dormant fibroblast cells highlights the accuracy of our experimental design since these cells are very sensitive to DNA damage and repair processes. This sensitivity is crucial for precisely evaluating the oxidative DNA damaging effects of Rutin and Naringin. This technique receives support from research conducted by Marthandan et al.^51^ and Chen et al.^52^, which emphasize the significance of choosing suitable cellular models for investigations.

Furthermore, the DDR's involvement in the survival of cancer cells and its dysfunction in malignant conditions present a potential opportunity for therapeutic intervention, which Rutin and Naringin seem to specifically target. Their capacity to specifically cause DNA damage in cancer cells might exploit the weaknesses of the DDR pathway, potentially resulting in the death of cancer cells. The data on synthetic lethality and the use of DDR inhibitors, such as PARP inhibitors, in the treatment of tumors with particular genetic abnormalities, as detailed in papers by^53,54^, provides support for this approach.

The lack of sufficient study on the oxidative DNA damage effects of Rutin and Naringin at a concentration of 10 µM in breast cancer cells highlights the originality of our findings and the necessity for more investigation. This investigation is crucial for unraveling the processes by which these chemicals exert their effects and for confirming their therapeutic potential.

The studies conducted by Al-Rajhi et al.^55^, Waleed Al-Areer et al.^56^, and Ghasemzadeh et al.^57^ support the idea that Rutin and Naringin can prevent or treat cancer. These investigations shed light on the chemicals' capacity to hinder the growth of cancer cells and trigger programmed cell death, emphasizing the need for additional research endeavors.

In addition to the sources, mentioned, other research has also contributed to our understanding of the anticancer characteristics of Rutin and Naringin. Cirmi^58^ propose that these flavonoids may have the ability to suppress mutagenesis, suggesting their potential as medications for treating cancer. Alam et al. Rahmat et al. focused on highlighting the investigated anticancer properties of these flavonoids found in the Rutaceae family^59,60^. In their report, Stabrauskiene et al. discuss the potential of Boesenbergia pulchella var attenuata as an anticancer agent, highlighting the presence of certain compounds. They suggest that these compounds could be used as supplementary remedies to suppress cancer development^61^. Additionally Egbuna et al. discuss the strong binding interactions between these compounds and the β-catenin protein, further supporting their potential as anticancer agents^62^.

The combined references highlight the potential of Rutin and Naringin as effective treatments for cancer, indicating the need for more study to understand their mechanisms of action and possible clinical uses.

## Conclusion

Based on our thorough analysis, the specific interaction between Naringin and Rutin with important DDR pathway proteins highlights their potential as natural regulators for cancer therapy. Their capacity to specifically attach to and possibly regulate the activity of proteins such as ATM, ATR, CHK1, and WEE1 reveals a pathway towards novel treatment approaches, emphasizing the essential role of DDR in the survival and growth of cancer cells. The study's findings indicate that the compounds examined had specific affinities for binding and interactions. This suggests a potential approach for modulating DDR and prompts additional investigation into how these compounds work within the intricate cellular environment of cancer.

The ADMET and drug-likeness study we conducted identified several limitations in the physicochemical features of Naringin and Rutin. However, it also emphasized their potential as drug candidates. These findings suggest the need for additional improvement to increase the absorption and efficacy of these medicinal compounds. Furthermore, the observed specific toxicity towards cancer cells, while having no impact on normal cells, confirms the promise of these chemicals to provide safer methods of treating cancer. The capacity of Naringin and Rutin to provoke substantial cytotoxic effects in cancer cells, without causing injury to normal cells, corresponds with the therapeutic imperative of selectively targeting cancer cells while maintaining the integrity of healthy tissue.

The evaluation of antioxidant activity using the DPPH test provides further evidence of the dose-dependent effectiveness of Naringin and Rutin, highlighting an additional aspect of their therapeutic capabilities. Their ability to reduce oxidative stress and prevent DNA damage not only expands their usefulness in preventative approaches but also in therapeutic interventions for illnesses connected to oxidative stress, such as cancer.

Our work has discovered a new and important result on the oxidative DNA damage effects of Naringin and Rutin, specifically their ability to selectively induce DNA damage in breast cancer cells. At a dosage of 10 µM, this phenomenon of selective DNA base and strand breaks provides an opportunity for further investigation into the processes that allow these natural substances to successfully differentiate between malignant and non-cancerous cells.

Our study identifies Naringin and Rutin as very promising natural compounds with substantial therapeutic potential, particularly in the field of cancer therapy. Their ability to modify DDR, selectively kill cancer cells, serve as antioxidants, and cause DNA damage in cancer cells highlights the importance of conducting more research to fully understand their mechanisms of action and possible uses in therapeutic settings. This endeavor, though challenging, has the potential to advance cancer therapy by developing more precise, efficient, and less harmful treatment approaches, utilizing the subtle healing abilities of natural substances.

## Data Availability

All data analysed during this study are included in this article. Further inquiries can be directed to the corresponding author.
